# Dry, rainfed or irrigated? Reevaluating the role and development of rice agriculture in Iron Age-Early Historic South India using archaeobotanical approaches

**DOI:** 10.1007/s12520-019-00795-7

**Published:** 2019-02-15

**Authors:** Eleanor Kingwell-Banham

**Affiliations:** Institute of Archaeology, University College London, London, WC1H 0PY UK

**Keywords:** *Oryza sativa*, Intensification, Urbanisation, South Asia, Phytoliths

## Abstract

Domestic rice agriculture had spread across the mainland Indian subcontinent by c.500 BC. The initial spread of rice outside the core zone of the central Gangetic Plains is thought to have been limited by climatic constraints, particularly seasonal rainfall levels, and so the later spread of rice into the dry regions of South India is largely supposed to have relied on irrigation. This has been associated with the development of ritual water features in the Iron Age (c.1000–500 BC), and to the subsequent development of tanks (reservoirs) during the period of Early Historic state development (c.500 BC–500 AD). The identification of early irrigation systems within South Asia has largely relied on early historical texts, and not on direct archaeological evidence. This initial investigation attempts to identify irrigated rice cultivation in the Indian subcontinent by directly examining rice crop remains (phytolith and macrobotanical data) from four sites. The evidence presented here shows that, contrary to accepted narratives, rice agriculture in the Iron Age-Early Historic South India may not have been supported by irrigated paddy fields, but may have relied on seasonal rainfall as elsewhere in the subcontinent. More caution is urged, therefore, when using terms related to ‘irrigation’ and ‘agricultural intensification’ in discussions of the Iron Age and Early Historic South Asia and the related developments of urbanism and state polities.

**Electronic supplementary material:**

The online version of this article (10.1007/s12520-019-00795-7) contains supplementary material, which is available to authorized users.

## Introduction

Within South Asia, the prehistory and history of rice has to be picked out using data points that are very few and far between (Bates et al. 2017; Fuller et al. 2010, 2016). Archaeology has now reached a stage where it is possible to detail where rice was first used as a major food resource (c.6400 BC in Uttar Pradesh; Tewari et al. 2008), to outline when it became a domestic crop in India (c.2500 BC; Bates et al. 2017; Murphy and Fuller 2016) and, roughly, when it spread across South India to reach Sri Lanka by c.500 BC (Fuller et al. 2010, 2016). However, we do not know how rice was grown in many areas of South Asia, when deep-water irrigation systems were developed or how the spread of rice outside of its natural zone was facilitated by human intervention and invention. By examining these questions, it is also possible to inform the development of state polities in South India, which is argued to have coincided with the introduction of deep-water irrigation and rice crops in these regions (Shaw and Sutcliffe 2003; Morrison 2015). Agricultural intensification in the form of irrigated rice has often been given a causal role in the development of urbanism (cf. Fuller and Qin 2009; Stargardt 2019; Wittfogel 1957). Across South Asia, and particularly Sri Lanka, irrigation tanks (artificial reservoirs of any size) form significant parts of the landscape, as well as cultural, agricultural and religious life (Shaw 2005; Sengupta 2000). The construction and maintenance of tanks (as well as other irrigation infrastructure such as canals and dams) requires community management, demands a high labour cost (Mosse 1999) and so their development and spread has been associated with the Early Historic development and spread of urbanism and Buddhism c.500 BC–500 AD (Shaw and Sutcliffe 2003; Morrison 2015; Morrison 2019). This theory, however, neglects other important factors in the development of states and urbanism, especially trade and economic specialisation (Allchin 1995; Johansen 2010; Moorti 1994; Smith 2006; Thakur 2002) and the autonomous agency of ‘ruled’ state subjects (cf. Hall 2001; Morrison and Junker 2002; Scott 2017). It also neglects alternative agricultural strategies such as diversification (Marston 2011; Petrie and Bates 2018; Weber et al. 2010a) and extensification (Porter et al. 2014; Strying et al. 2017).

This paper presents archaeobotanical data from four sites separated by up to 3000 km and 3000 years, from the north to the south of India: Tokwa, Gopalpur, Perur and Kodumanal. This analysis aims to directly investigate the ecology of the rice crops cultivated at each site, via archaeological phytolith samples (silica bodies which form within and between plant cells), in order to confirm the emergence of irrigated cultivation systems in the Early Historic South India.

## Rice, rain and reservoirs

Rice agriculture is intrinsically coupled to the monsoons in South Asia, which provides the bulk of water used in agriculture. The southwest summer monsoon begins in June, reaching Sri Lanka and the south of India first and ending in the northwest of India in September. Caused by low pressure over the subcontinent and the high wall of the Himalayas, winds bring moisture from the Indian Ocean in huge amounts, leading to rainfalls of up to 2500 mm in just 2 months on the southwest coast and northeast India. The northeast monsoons take place between October and February and are the result of the Indian subcontinent cooling after the summer months. This monsoon brings water to the east coast, areas of Central India and Sri Lanka, and accounts for around 50% of Tamil Nadu’s annual rainfall (Fick and Hijmans 2017). The period between the two monsoons, summer, sees very high temperatures and little to no rainfall in India. There are, therefore, three seasons across most of India: monsoon, summer and winter (during the northwest monsoon). Some coastal parts of southern India have a different pattern of four seasons, with the southwest monsoon from June to October, the northeast monsoon from December to March, and two intermonsoonal periods. This causes a different pattern of vegetative growth from the rest of India, with flowering occurring in the winter, rather than the monsoon season (Asouti and Fuller 2007). The scheduling of crop growing seasons is, naturally, heavily influenced by the monsoons (see discussion in Morrison, in press). In India, there are two growing seasons: kharif (coinciding with the summer monsoon) and rabi (coinciding with the northeast monsoon). Both are timed so that planting coincides with the beginning of the rains, and harvesting occurs several weeks after they have ended. Traditionally, across most of India, the kharif crop is characterised by rice, millets and *Vigna* pulses, whereas wheats, barley and peas are grown as the rabi crop. Not all areas can produce a double crop; today, the rabi crop is largely found in the states of Tamil Nadu, Kerala, Telangana and Andhra Pradesh, as well as across soils of Indus-Ganges alluvium, and is facilitated by the use of irrigation technologies. The production of kharif and rabi crops is not solely dependent on the monsoon rains however. Irrigation technologies allow for the production of multiple crops (including double or even triple rice cropping (Petrie and Bates 2017)) in areas that would not necessarily receive enough rainfall (for example, by pumping ground water). Local environments and geographies also play a role, such as in the Kaveri delta in Tamil Nadu where multiple cropping is supported by the waters of the delta.

Of particular importance around the early first millennium AD were tanks and canals. References to such irrigation works are frequently recorded in Sangam texts of South India and it is clear that undertaking irrigation projects was a noble and lofty scheme for kings to undertake (Ramaswamy 2008; Raman 2008). Dating these texts, particularly the earliest, remains problematic, however, with a reasonable estimate of 300 BC–300 AD proposed by Abraham (2003 p. 214) and Thapar 2004 p. 231) for the Early Sangam period. Direct dates on tank construction are so far unavailable in South India, but the initial construction phase for a tank in the Anuradhapura region of Sri Lanka has been placed at c.400–200 BC using Optically stimulated luminescence dating (Gilliland et al. 2013). It is suggested that this tank was used for small-scale agriculture within the developing urban hinterland of Anuradhapura City (ibid.). The earliest direct dates for rice from Sri Lanka come from Kantharodai c.300 BC and Mantai at 74–241 cal AD (Kingwell-Banham et al. in press).

Whilst irrigation technologies allow for a degree of mitigation against both drought and flooding, the quantity, intensity and availability of rainfall were of greater importance in pre-irrigation technology agriculture than they are today. The earliest rice-producing societies all occur within areas that receive substantial monsoon rainfall and seasonal flooding: the Indo-Gangetic basin and East India (Kingwell-Banham et al. 2015). Across the semi-arid areas of South Asia agricultural production focused on drought tolerant millets and pulses as well as wheat and barley, which formed the basis of crop production in Neolithic South India c.2800–1000 BC (Fuller 2006; Kingwell-Banham et al. 2015). Wheat and barley, whilst needing more water than millets, still require less water than rice in predominant cultivation systems. This pattern suggests that a change must have occurred to either the environment, agricultural systems (including through technological innovation) or to the rice crop itself, before rice agriculture could move from within its restricted environmental sphere into the drier areas of South Asia around 500 BC.

Unfortunately, palaeoecological studies for the Deccan and central South India are rare. What little data that exists suggests that monsoon variability increased in the Late Holocene, but that this had different regional expressions (Patnaik et al. 2012; Ponton et al. 2012; e.g. compare Prasad et al. 2014 to Tripathi et al. 2014). Roberts et al. (2016) have examined changes in settlement patterns, subsistence and demographics in relation to environmental change during the Late Neolithic-Megalithic transition in Bellary, Karnataka. This study has suggested that fluctuations in rainfall levels would have made access to reliable watercourses, such as the Krishna and Tugabhadra rivers, of great importance. This is demonstrated by the increase in settlement size and density along such watercourses within the Southern Deccan and the abandonment of settlements located away from rivers, such as Sanganakallu and Hiregudda Area A (Roberts et al. 2016 pp.596–5). Similar shifts in settlements in the Vijayanagara region have been reported by Morrison (2015 p. 12) for the Late Iron Age-Early Historic period, although she did not identify such change during the Neolithic-Iron Age period. It is unlikely that monsoon variability would have caused a large enough increase in rainfall to allow for significant non-irrigated wet-rice agriculture in central South India c.1000 BC onwards. It appears, however, that human response to this variability included concentrating settlements along larger rivers and tracts of alluvium that could have provided adequate water for crop production (Roberts et al. 2016).

## The development of rice agriculture in South Asia: established narratives and gaps in the data

The use of archaeobotanical data to investigate the impact of monsoon variability on agriculture in South Asia has been hampered by the fact that few sites have been systematically sampled for archaeobotanical remains, and fewer of these have been published. This is particularly true for South India (Table 1). Yet, there is not enough data to fully examine changes in crop composition between the Neolithic-Iron Age-Medieval periods; however, it is possible to trace an outline.

**Table 1:** Key crops of South India, their earliest reported dates and the number of sites they have been reported. Data from Stevens et al. (2016), Supplementary Table 1, which records published archaeobotanical data and associated radiocarbon dates. Note the reduction in published archaeobotanical data sets from post-Neolithic sites (1000 BC onwards)

Area of origin	Crop	Earliest reported date in South India (BC)	Number of sites present at 2000–1000 BC	Number of sites present at 1000–500 BC	Number of sites present at 500–0 BC	Number of sites present at 0–500 AD
South Asia	*Macrotyloma uniflorum*	1900	17	2	3	
South Asia	*Vigna radiata*	1875	10	3		
West Asia	*Triticum aestivum*	1800	7	2		
West Asia	*Hordeum vulgare*	1800	7	1	1	
West Asia	*Lathyrus sativus*	1800	2			
South Asia	*Panicum sumatrense*	1800	2	1	2	
Africa	*Paspalum scrobiculatum*	1800	5		2	
South Asia	*Cajanus cajan*	1800	4	1		
South Asia	*Setaria verticillata*	1750	9			
South Asia	*Brachiaria ramosa*	1700	15	2	1	
South Asia	*Vigna mungo*	1650	5		2	
Africa	*Pennisetum glaucum*	1550	2	1	1	
South Asia	*Oryza sativa indica*	700		3	13	3
Africa	*Sorghum bicolor*	550		1		
Africa	*Vigna unguiculata*	550		1		
		Total	85	18	25	3

**Fig. 1:**
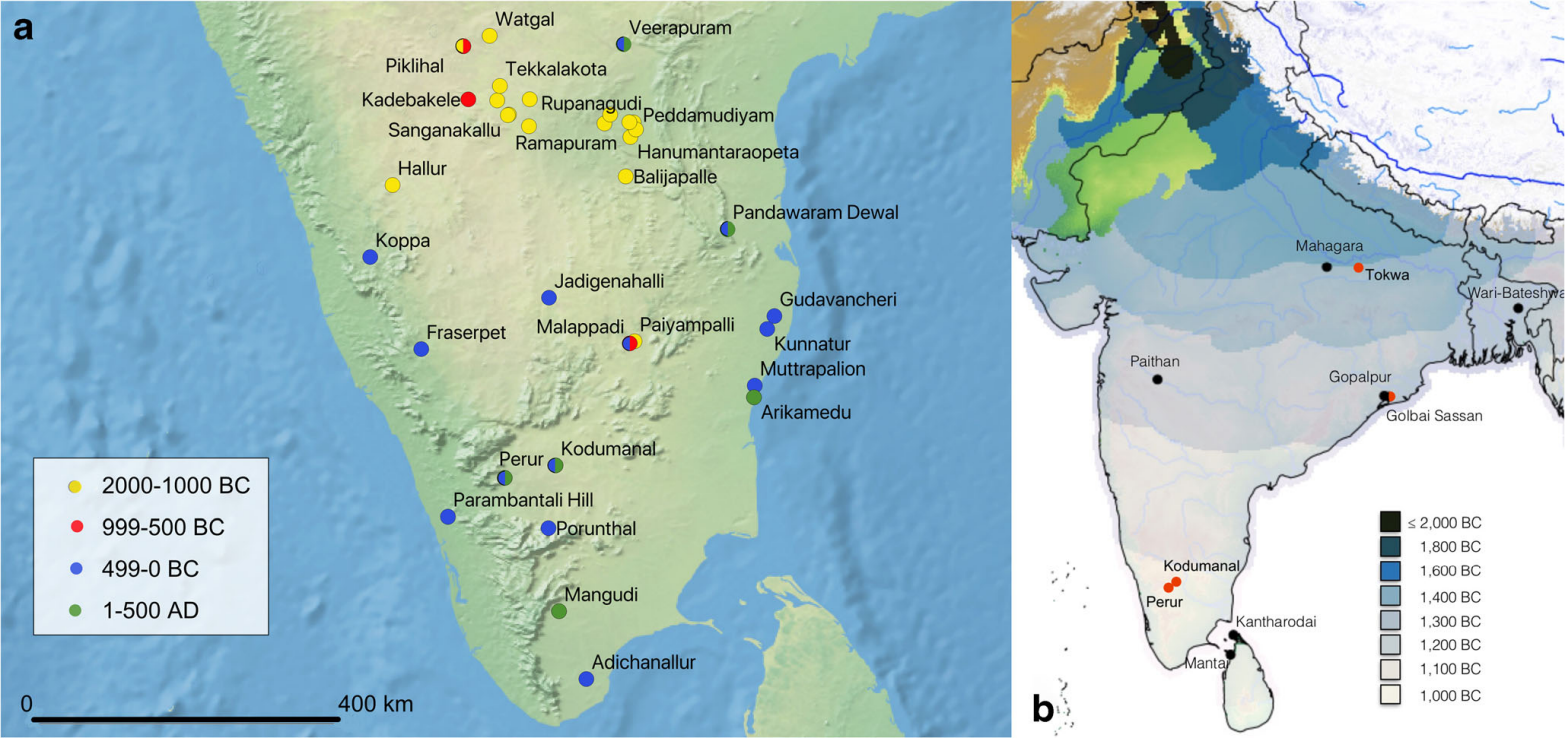
**a** Locations and time periods of archaeological sites in South India with published archaeobotanical data recorded in Stevens et al. (2016). **b** Map showing the spread of rice across South Asia based on Fast-March modelling, from Silva et al. (2018). Sites in black have macrobotanical remains of domesticated-type rice spikelet bases; sites in red are those studied in this article

There is a period of approximately 1000 years in which the transition from using proto-*indica* rice (the early rice variety indigenous to India) to supplement subsistence in the Ganges Basin to the use of rice as a primary cultivated domestic crop across the Indo-Gangetic Basin occurred, c.2500 BC (Silva et al. 2018). There is a hiatus, however, before rice cultivation is taken up in drier parts of India: the Deccan and South India (Fig. 1). It is not until the second half of the first millennium BC that we see archaeobotanical evidence and rice-tempered pottery suggesting the use of rice as a major crop (Fuller and Qin 2009; Fuller 2006). This final dispersal of *Oryza sativa* in India has been attributed in part to the spread of Buddhism and irrigation technology in the area between the 3rd–1st centuries BC (Shaw et al. 2007; (although others have dated this more recently, e.g. Raman (2008 p. 497) at mid-first millennium AD), but has also been given causality in the rise of urbanism (e.g. Fuller and Qin 2009).

The spread of rice agriculture into the savannahs of southern India is postulated to have been via the eastern coast (Cooke et al. 2005), but may equally have travelled along the western coast as the data are very patchy. Both the coastal regions to the sides of the Western and Eastern Ghats see higher levels of rainfall than the central zone during the summer monsoon and so could have supported a rainfed rice crop. It is also possible that early forms of saltwater/saline rice cultivation systems (e.g. kaipad) may have developed in these regions, but this remains speculation. From there, either before or after the development of irrigation systems, rice cultivation may have moved over these mountain ranges and into the interior, possibly along rivers such as the Krishna or Godavari and their tributaries (see Raman 2008 for notes on the importance of riverside agricultural land in the Sangam period). By 1000 AD urban centres, tanks and irrigation networks were present across arid South India. Undoubtedly, by this time, irrigated agriculture was important in supporting larger urban populations and irrigated cash crops such as sugarcane and cotton became increasingly important to the economy (Thakur 2002; Fuller 2008; Fuller et al. 2017).

## Intensification versus extensification

The leading theory as to what allowed rice agriculture to move into the dry zones of the Southern Peninsula does not primarily come directly from archaeobotanical analysis, but from the archaeologies of settlement, landscape, state development and from the studies of early historical texts. This theory ties the spread of rice agriculture to the development of irrigation technologies, the construction of tanks, ponds and canals across the Deccan, the Southern Peninsula and Sri Lanka at the end of the Iron Age/beginning of the Early Historic Period, and the development of early polities and urbanism (e.g. Shaw and Sutcliffe 2003; Bauer and Morrison 2008; Gilliland et al. 2013) and can be traced back to Wittfogel’s seminal work (Wittfogel 1957).

Irrigation is a form of agricultural intensification which can dramatically increase yields (Boserup 1965; Brookfield 1972; McClatchie 2014), and as such is often associated with the development of densely populated urban societies across the world (e.g. Marcus and Stanish 2006; Weiss 1986); however, this is a simplified understanding of the dynamics between agriculture and populations (e.g. Erickson 2006; Kirch 1995; Morrison 1994). Other forms of agricultural intensification include manuring and weed management, both of which can be analysed archaeobotanically (see Jones et al. 2000; Bogaard et al. 2005), but these have often been overlooked in South Asian archaeobotany in favour of irrigation, undoubtedly because deep-water irrigated paddy fields are visually conspicuous within the modern landscape. Flooded and transplanted rice cultivation systems (which the term ‘paddy’ most often relates to, when not used more generally simply mean ‘rice’) are almost unique in that the process of flooding fields and transplanting seedlings both fertilises and dramatically reduces the amount of weeding needed to raise a crop. It is a highly labour intensive system of cultivation (including the creation of bunds [linear banks of earth], canals, damns and sluices) but with some of the highest yields. Compare modern yields of deep-water irrigated rice of around 2.5 t/ha to around 1 t/ha for rainfed rice (IRRI 2000), for example. The early development of irrigated fields has been documented archaeologically in Neolithic China, where small, enclosed, rainfed ‘ponds’ developed into large canal fed paddy fields (Fuller and Qin 2009; Zhuang et al. 2014), but not in South Asia.

Intensification is not, however, the only way in which past societies increased crop yields. Diversification of crops (see Marston 2011), including the establishment of summer and winter cropping, has frequently been considered within the archaeology of Northern India in particular (e.g. Petrie and Bates 2017; Weber 1998; Weber et al. 2010a) and to a more limited extent in South India (e.g. Cooke and Fuller 2015). *Extensification* has been greatly overlooked in the research of the Early Historic South Asia however (although see Miller 2006). Extensification refers to the process of increasing crop production by bringing more land under cultivation. Obvious indicators of extensification include deforestation (most often seen archaeologically through palaeoclimate records as increased microcharcoal or decreased arboreal pollen, e.g. Penny and Kealhofer (2005)) and the creation of new fields and field boundaries over a large area (e.g. Porter et al. 2014). McClatchie (2014) convincingly argues against oversimplification in the identification of ‘intensive agriculture’ through the creation of stone-built field boundaries. These have been interpreted as the establishment of fixed plot, more intensive agriculture, developing from less intensive systems of shifting cultivation. As McClatchie details, however, ‘a change in the organisation of production … does not necessarily imply any enhancement in productivity’ and instead may mark changes in the conceptual and social demarcation of landscapes. This can be related to Bauer and Morrison’s (2008) consideration of the socially symbolic importance of early reservoirs in South India. These changes in social landscape may include the process of extensification, as a social group incorporates larger areas into their agricultural territory, such as the Tiv’s extensive agriculture in the Benue Lowlands. As Stone (1996 p. 189) describes it ‘in location after location, land pressure brought not the heightened work of intensification, but movement’, indicating that extensive agriculture can sometimes provide a more resilient and productive option. This resilience is demonstrated in the continued existence of shifting cultivators within India today, who instead of being seen as marginalised and ‘pushed out’, instead could be seen as groups who have maintained their cultural and economic identities despite consistent pressures to ‘modernise’ (Guha 1999; Kingwell-Banham and Fuller 2012; Morrison 2007; Scott, 2009, b).

Within South Asian archaeology, there tends to be an assumption that the presence of rice = presence of irrigation = intensive agriculture and centrally controlled labour = increased production and political control = urbanism. Yet, however, there is unfortunately little archaeobotanical evidence to support this assumption outside of the comparatively well-researched Harappan Civilisation (Miller 2006; Weber 1999; and for replies, see Fuller 2001; Weber et al. 2010a) and studies from around the world into agriculture, intensification and the emergence of complex societies repeatedly demonstrate that models like this are too simple. Challenges to this and similar models have been proposed for places as disparate as the Indus Civilisation (e.g. Miller 2006, who critically reanalyses the Wittfoglian hypothesis; Petrie and Bates, 2017, who discuss intensification via multi-cropping), Africa (see Connah 2001, who also provides an overview of state formation theories), the Early Historic Mesopotamia (Styring et al. 2017, who show evidence for extensive agriculture in an early urban environment), China (e.g. Liu 2005 for the importance of craft specialisation, bronze and other non-agricultural resources in the development of urbanism) and from, of course, the complex hunter-gatherer societies of the Americas (Ames 1994; Fitzhugh 2003; Marquet et al. 2012, who document non-agricultural complex societies). Challenges to the idea that centralised government was needed for tank construction have also been recently made for South India, with Stargardt (2019) suggesting a bottom-up management of irrigation infrastructure in the first millennium AD. It is clear, therefore, that more care needs to be applied when interpreting the relationship between rice agriculture, irrigation, intensification, state formation and urban complexity in South Asia. To this aim, a preliminary study into the agricultural field systems of ancient South Asia was conducted. This article presents the first attempt to directly identify crop irrigation within the archaeological record of South Asia using the crop remains themselves.

## Materials and methods

### The sites

**Fig. 2:**
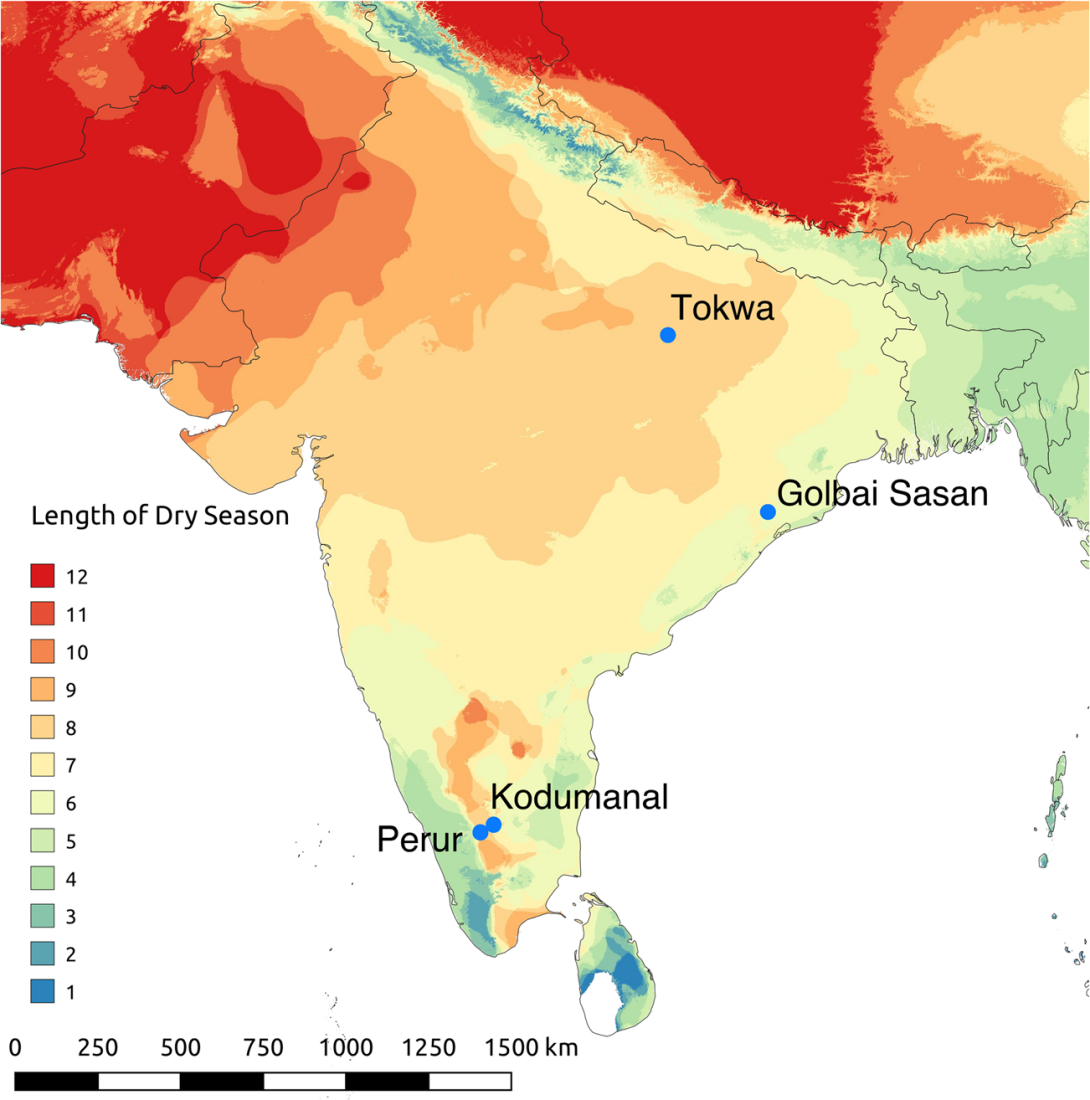
Map of South Asia showing the location of sites studied in this paper and length of dry seasons (based on the average monthly precipitation data in WorldClim v2 (Fick and Hijmans 2017). Dry season months were identified where monthly precipitation was below 60 mm, as per the Köppen climate classification system (Koppen 1923)). (Map made by F. Silva)

Four sites were analysed from three separate archaeological and cultural periods, and radiocarbon dates were taken directly from rice grains recovered within the material analysed here (Fig. 2; Table 2).

**Table 2:** Published dates for Tokwa, Golbai Sasan, Perur and Kodumanal (Kingwell-Banham 2015)

Site	State	Time period	Date cal BC/AD
Tokwa	Uttar Pradesh	Neolithic-Chalcolithic	2500–1000 BC
Golbai Sasan	Odisha	Neolithic-Chalcolithic	1400–900 BC
Perur	Karnataka	Early Historic	260–557 AD
Kodumanal	Karnataka	Early Historic	430–230 BC

Tokwa is a Neolithic-Chalcolithic site situated in the Uttar Pradesh on the confluence of the Belan and Adwa rivers (Misra et al. 2000–2001). Uttar Pradesh receives around 80% of its annual rainfall during the summer monsoon (WorldClim v2 data, Fick and Hijmans 2017). It is associated with several other Neolithic-Chalcolithic sites, dated to c.2500–1000 BC, with evidence of early permanent settlement and agriculture, including Koldihwa, Mahagara and Senuwar. These sites all have evidence for the production of summer (rice) and winter crops (wheat, barley), indicating the presence of two agricultural seasons per year (Kingwell-Banham 2015; Pokharia 2008) (Table 3). Golbai Sasan is a Neolithic-Chalcolithic site of East India, located in Odisha, close to Chilka Lake and on the bank of the Mandakini River. This area also receives the majority of its annual rainfall during the summer monsoon. Golbai Sasan is dated to c.1400–900 BC and rice and pulses have been recovered from the site, indicating that agricultural production was focused on summer crops (Harvey 2006; Kingwell-Banham 2015; Kingwell-Banham et al. 2018). Tokwa and Golbai Sasan are both situated in low-lying floodplain environments that receive adequate rainfall during the summer monsoon to produce good yields of rice without supplemental irrigation. Phytolith remains from these sites will therefore produce a clear-rained rice signature against which to compare the data from Perur and Kodumanal.

**Table 3:** Ubiquities of main crops identified at Tokwa, Golbai Sasan, Perur and Kodumanal (Cooke et al. 2005; Kingwell-Banham 2015)

Site	Rice (% ubiquity)	Wheat and Barley (% ubiquity)	Small millets (% ubiquity)	Pulses (% ubiquity)
Tokwa	67	57	0	86
Golbai Sasan	50	0	19	10
Perur	100	0	50	60
Kodumanal	60	0	60	90

Perur and Kodumanal are both located in Tamil Nadu, a much drier state than Uttar Pradesh and Odisha, which receive the majority of its rainfall during the winter monsoon. Both sites belong to the Early Historic-Medieval period. Rice from Kodumanal has recently been dated to 430–230 BC and Perur from 260 to 557 AD (Table 2). Kodumanal is situated further inland than Perur, which benefits slightly from the increased precipitation of the Western Ghats; thus, Kodumanal receives less rainfall than Perur (Fig. 2). Rice, tuber parenchyma and some millets were recovered in the macrobotanical remains from both sites. In addition, pulses were recovered from Kodumanal and Perur (Cooke et al. 2005) (Table 3).

A common problem in Indian archaeobotany is that the preservation of macrobotanical charred remains is often poor. This particularly affects less robust plant remains such as chaff and some of the smaller seeds of weedy plants, creating differential preservation between these items and cereal grains and pulses. Recovery practices are also implicit in this, with samples floated onto a 0.5-mm sieve less likely to contain small seeds of weedy plants than those floated on a 0.25-mm sieve (e.g. compare Tokwa [0.5-mm sieve] to Golbai Sasan [0.25-mm sieve] in Table 4). As a result, it is often a challenge to use weedy taxa as ecological indicators. This means that it is very difficult (in many cases impossible) to reconstruct crop agricultural practices such as manuring, field rotation or ploughing, or irrigation using macrobotanical data. The weedy seed assemblage for each site analysed here is presented in Table 4. Of note is the low number of species level identifications across the sites, even when the assemblage is relatively large as at Golbai Sasan. Therefore, other means of investigation must be used, and phytolith analysis in particular has proven to be a strong tool in the identification of irrigated field systems.

**Table 4:** Macrobotanical data for weedy plants recovered from Tokwa, Golbai Sasan, Kodumanal and Perur (Cooke et al. 2005; Kingwell-Banham 2015). *Fimbristylus* sp. (x) reported by Pokharia 2008

	*Tokwa*	*Golbai Sasan*	*Kodumanal*	*Perur*
Period	Neolithic	Neolithic-Chalcolithic	Early Historic	Early Historic
# of samples	18	44	10	10
Total volume (L)	Unknown	696	200	200
cf. Aizoaceae			3	18
Aizoaceae		1		
cf. Araliaceae		3		
cf. Asteraceae				1
Asteraceae		3	1	
Asteraceae *Tridex* type		1		
*Bromus ramosus*	2			
Boraginaceae			2	
*Chenopodium* sp*.*	1			2
Commelinaceae				1
cf. Cruciferae				1
cf. *Crotolaria* sp.				2
cf. Cyperaceae			5	20
Cyperaceae		6		
Euphorbiaceae				3
*Fimbristylus* sp*.*	x			
*Ischaemum rugosum*				7
cf. *Ipomea* sp.		1		
Lamiaceae		2		
cf. Liliaceae		3		
cf. *Lolium* sp.	1			
Malvaceae		3	2	2
cf. *Molluga* sp.			2	
cf. *Oldenlandia* sp.		1		
cf. *Phyllanthus* sp*.*		1		
Polygonaceae		1		
*Portulaca* sp*.*		1		
Rubiaceae		2	1	1
cf. *Rumex*		1		
cf. *Lindernia/Scropia* sp.		4		
cf. *Schoenoplectus*		6		
*Sida* sp*.*		1		
*Sisyrinchium* sp*.*		5		
cf. *Scirpus* sp.		2		
cf. *Stellaria* sp.			1	
*Verbascum* sp*.*				7
Wild seed indet.		13	8	27

### Identifying rice cultivation systems

Different field management and cultivation systems produce different field ecologies, and this has been used in archaeobotany to identify cultivation practices and changes to cultivation practices over long- and short-time frames, from more simple analyses of the abundance of certain weed-type floras (e.g. Jones 2009), to more complex analyses of weed-type floras (e.g. Bogaard et al. 1998; Jones et al. 2000) and phytolith assemblages (e.g. Weisskopf et al. 2013). Due to the relative sparsity of the macrobotanical weeds within the assemblages from each of the sites investigated, analysis of the phytolith data has provided the main avenue of investigation.

**Fig. 3:**
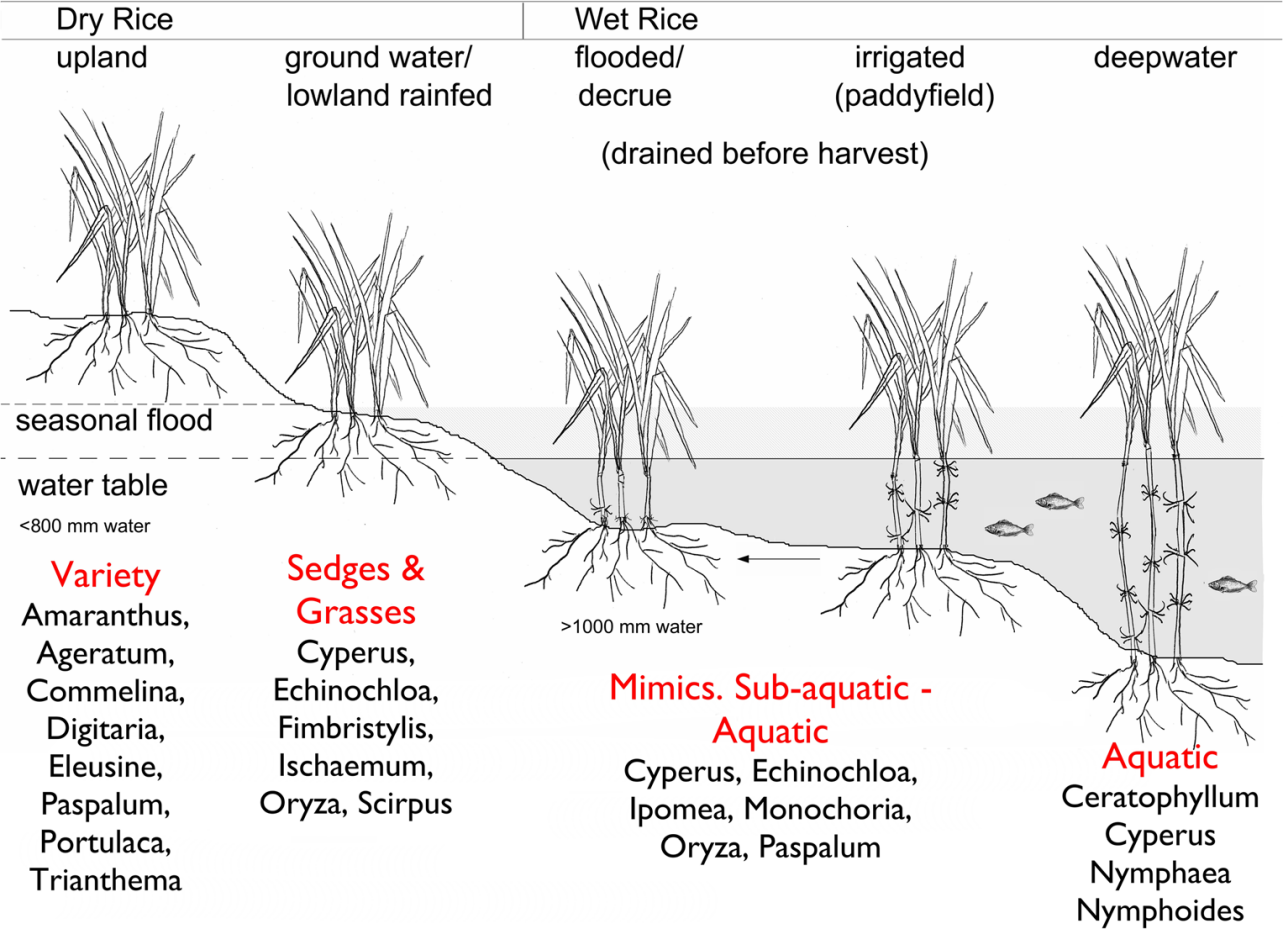
Rice cultivation systems classified according to elevation and water availability, with example weed profiles (as per Fuller et al. 2010)

The variation in methods to both irrigate crops and cultivate rice can make it difficult to develop clear classifications for the difference between, for example ‘dry’ and ‘rainfed’ crops, or ‘wet paddy’ and ‘irrigated’ rice. With regard to human agency, irrigation is defined as any artificial process which increases the supply of water to land with growing crops (Dictionary of agriculture, third edition, 2011). In order to distinguish between different cultivation systems archaeobotanically, rice cultivation systems were defined based on water availability during the growing season by UCL’s Early Rice Project (Fuller et al. 2010). The rationale behind this is that the high-yielding irrigated rice that is of particular significance in archaeological narratives require a very high quantity of water, that is (with the exception of some forms of decrue cultivation) above the level of naturally available seasonal rainfall across Asia. This water-based definition allows for differentiation between systems based on their ecological signatures and associated wild weedy floras (Fig. 3). As such, ‘dry cultivation’ refers to rice that was cultivated with < 800 mm of water over the growing season, ‘wet cultivation’ with > 1000 mm of water over the growing season. Rainfed cultivation systems can be found along the spectrum from 800 mm +, and can fall into the category of ‘wet cultivation’ alongside irrigated rice. There is ambiguity with regard to cultivation systems using between c.600–999 mm of water, i.e. are they truly ‘dry’ or ‘wet’/irrigated or rainfed. Archaeobotanically, cultivation systems falling towards the middle of the spectrum are very hard to differentiate between as they will have similar field ecologies and weed floras. It is therefore necessary to consider other forms of evidence alongside the archaeobotanical data in order to identify these cultivation systems. For the time periods under investigation at Tokwa and Golbai Sasan, for example it is possible to be clear that the crops were rainfed, as opposed to irrigated, due to the absence of irrigation technology at that time. Both sites are in areas with good monsoon rainfall and will produce a signal for ‘wet’ rice cultivation against which the results from Kodumanal and Perur can be compared.

In order to identify irrigation, phytoliths with a rate of production determined by water availability were specifically pinpointed within the analysis of the results. ‘Fixed’ morphotypes (grass phytoliths with a rate of production that is not related to water availability), ‘sensitive’ morphotypes (grass phytoliths that have a rate of production directly correlated with water availability) and ‘other grass multi-cells’ (grass multi-celled phytolith panels) were separately classified (Table 5). In grasses, the long cells and stomatal cells (sensitive morphotypes) which show a quantifiable difference in production in *Triticum aestivum*, *Triticum dicoccum*, *Hordeum vulgare* and *Hordeum aegiceras* grown in controlled dry and wet conditions (Madella et al. 2009) and *Triticum durum* and *Hordeum vulgare* grown in agricultural fields (Jenkins et al. 2011, 2016). This pattern has been demonstrated to apply to rice using modern field samples from India (Weisskopf et al. 2015) and China (Huan et al. 2018), as well as archaeological samples from both field and cultural contexts in Neolithic China by Weisskopf et al. 2015). ‘Sensitive’ morphotypes have been proven to be represented in higher proportions from archaeological sites with irrigated rice cultivation systems compared to drier rice cultivation systems (Weisskopf et al. 2015). The sites included in this study were Tianloushan (wetland decrue), Caoxieshan (small, frequently drained fields) and Maoshan (large, intensive, irrigated fields). A sensitive to fixed phytolith index (where a higher value = wetter growing conditions) correlated with the archaeological, geoarchaeological and macrobotanical evidence for field cultivation systems, with Tianloushan showing 2.5, Caoxieshan showing 0.7 and Maoshan showing 2.0. Additionally, the production of multi-cell panels (conjoined cells) appears to occur more frequently in *Triticum dicoccum*, *Triticum aestivum*, *Hordeum vulgare* and *Hordeum spontaneum* grown in wet conditions (Jenkins et al. 2011; Rosen and Weiner 1994). However, the processes of disarticulation in archaeological contexts are not clear (Jenkins et al. 2011 p. 370), and my personal observations suggest that there may be an increased rate of post-depositional disarticulation in more water-rich environments. Nevertheless, ‘other grass multi cells’ were categorised separately, as potential indicators of wet land environments (based on Jenkins et al. 2011 and Rosen and Weiner 1994), but also as potential indicators of dryland cultivation systems (as per Fig. 3). Cyperaceae are more prevalent in wet cultivation systems than dry cultivation systems, although are by no means exclusive to wet ecologies. Dicotyledons are more prevalent within dry cultivation systems, e.g. *Amaranthus* sp., and grasses tend to be more prevalent within the middle of the water-availability spectrum (such as rainfed systems), although again this is not exclusive. Examining these different categories of data in parallel, as opposed to focusing on a single category, should allow for a comprehensive analysis of the results.

**Table 5:** Categories of phytoliths used in correspondence analysis

Rice	Hydrophilic	Cyperaceae	Fixed	Sensitive	Other grass multi-cells	Dicotyledon
*Oryza* bulliform cf. Oryza *bilobe* Leaf/culm *Oryza* cf. Oryza *husk*	Bulliform *Cuneiform bulliform* Leaf/culm Phragmites *Leaf/culm reed* Leaf/culm square cell	Long rods *Cones* Sedge achene cells *Cyperaceae leaf* Cyperaceae husk	All grass short cells (bilobate, rodel, trapezoid, crenate, cross, etc.)	All grass long cells (smooth, sinuous, dendritic)	Indeterminate leaf/culm *Leaf/culm bilobate* Leaf/culm cross *Leaf/culm rondels* Leaf/culm saddles *Leaf/culm long cells* Indeterminate husk cf. Setaria *husk* cf. *Panicum* husk *Millet type 1* Millet type 2	Smooth spheroid *Platey* Perforated sheet *Single polyhedron* Scalloped *Single jigsaw* Polyhedral hair base *Multi polyhedrons* Two-tiered *Multi-tiered*

### Sampling, processing and identification of phytoliths

Phytolith samples were collected at 10-cm intervals from a single trench section that spanned the entire chronological sequence of the site at Golbai Sasan. At Tokwa, Perur and Kodumanal phytolith samples were collected from the same stratigraphic layers as macrobotanical samples. Phytolith samples were processed at the UCL Institute of Archaeology using the heavy liquid separation method developed by Arlene Rosen (see Piperno 2006). 0.8 g of fine sediment was processed per sample and samples were mounted to slide using Entellen. Identifications were done using a biological microscope with a cross-polarising filter at × 400 magnification. A minimum of 300 single cells and 100 multi-cell panels were counted per sample, when possible. Identifications were made using reference slides and relevant literature (for example Eichhorn et al. 2010; Lu et al. 2009; Piperno 2006). Analysis of the phytolith data was done using correspondence analysis, which has been shown to provide an effective means to distinguish between different rice cultivation systems across Asia (Weisskopf et al. 2013; Weisskopf 2017). Canoco for Windows 4.5 and CanoDraw for Windows 4.1 (ter Braak & Smilauer 1998) were used to analyse and plot the data.

## Results

**Fig. 4:**
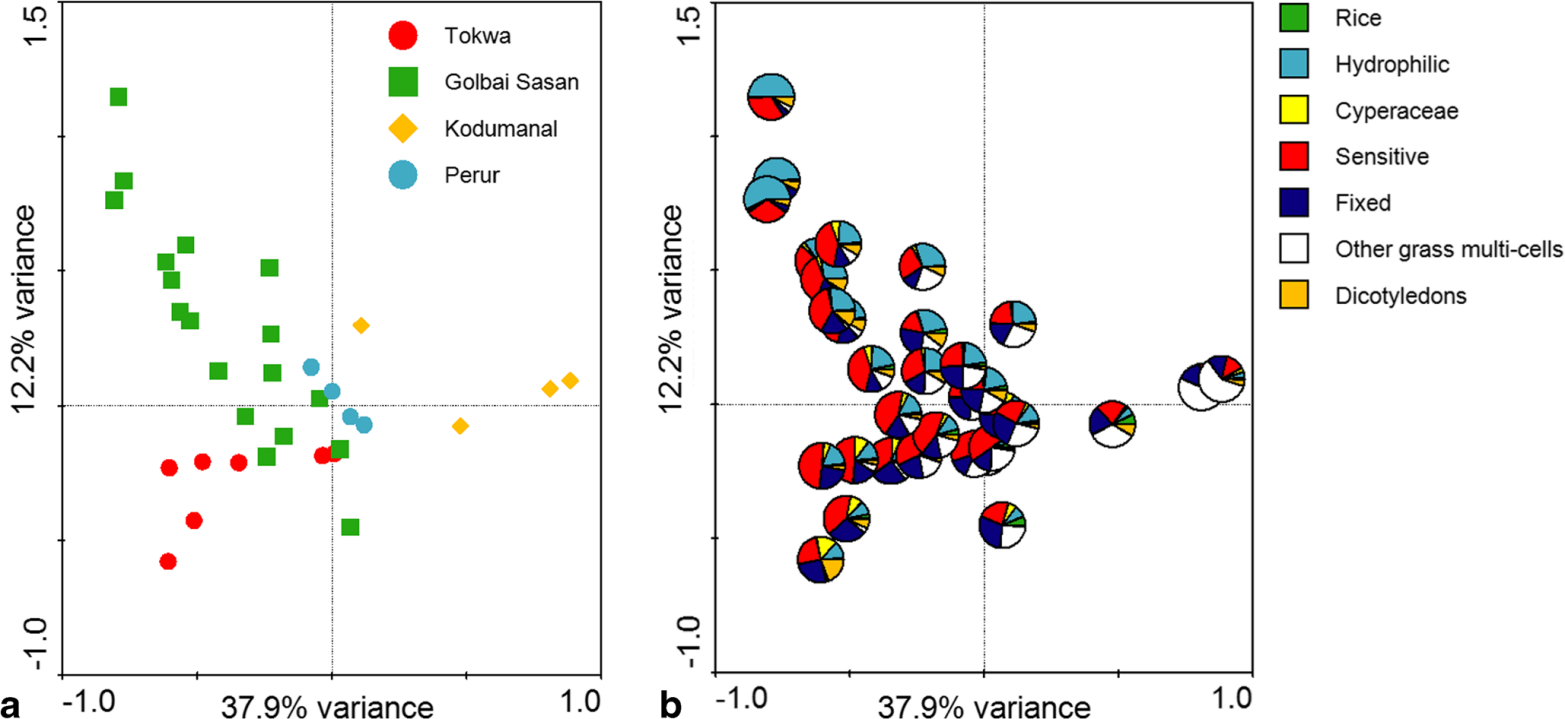
Correspondence analysis plots of phytolith data from Tokwa, Golbai Sasan, Kodumanal and Perur. **a** Samples classified by site. **b** Samples represented as pies, classified according to Table 4

Figure 4 shows the results of the correspondence analysis. In Fig. 4 a, a clear separation between the samples from Kodumanal (located towards the positive end of the *X* axis), Golbai Sasan (located towards the positive ends of the *X* and *Y* axes) and Tokwa (located towards the negative ends of the *X* and *Y* axes) can be seen. The samples from Perur plot closely together towards the middle of both axes.

Figure 4 b shows the same plot; however, each sample is represented by a pie chart. The pie charts are colour coded according to the classifications of phytoliths in Table 4. This figure shows that samples plotted towards the positive end of axis *X* have a higher quantity of ‘other grass multi-cell’ phytoliths and that samples plotted towards the negative end of axis *X* have a higher quantity of ‘hydrophilic’ phytoliths. Along axis *Y* samples with a higher quantity of ‘fixed’ and ‘Cyperaceae’, morphotypes are plotted towards the negative end. In general, samples with a more equal ratio of fixed to sensitive morphotypes and a lower frequency of hydrophilic morphotypes are plotted at the positive end of axis *X*, on the right hand side of the chart. This includes all of the samples from Kodumanal and Perur, as well as three samples from Gobai Sasan and two from Tokwa. On closer examination, these samples from Golbai Sasan and Tokwa have a higher proportion of sensitive to fixed morphotypes. Table 6 shows the ratio of sensitive to fixed morphotypes from each site and demonstrates that Kodumanal and Perur have lower values than Golbai Sasan and Tokwa.

**Table 6:** Sensitive to fixed morphotypes ratios for Tokwa, Golbai Sasan, Perur and Kodumanal

	Tokwa	Golbai Sasan	Kodumanal	Perur
S:F	2.17	2.03	1.12	1.25

## Discussion

The results indicate that the rice at Kodumanal and Perur was grown in an environment with lower water availability than at Tokwa and Golbai Sasan. Kodumanal and Perur both have higher quantities of ‘other grass multi-cells’ to dicotyledon phytoliths, suggesting a higher proportion of grass weeds (and that these correlate with dryland cultivation system ecology and not water availability), as well as lower sensitive to fixed ratios and a lower quantity of Cyperaceae phytoliths, suggesting lower water availability during the growing season. This is consistent with a ground water/lowland rainfed cultivation system (Fig. 3). Conversely, the samples from Golbai Sasan and Tokwa show a high quantity of sensitive morphotypes and dicotyledon phytoliths. Golbai Sasan in particular shows a very high quantity of hydrophilic morphotypes. This is consistent with wet-rice agriculture (Fig. 3, ‘flooded/decrue’, ‘irrigated’ or ‘deepwater’).

**Fig. 5:**
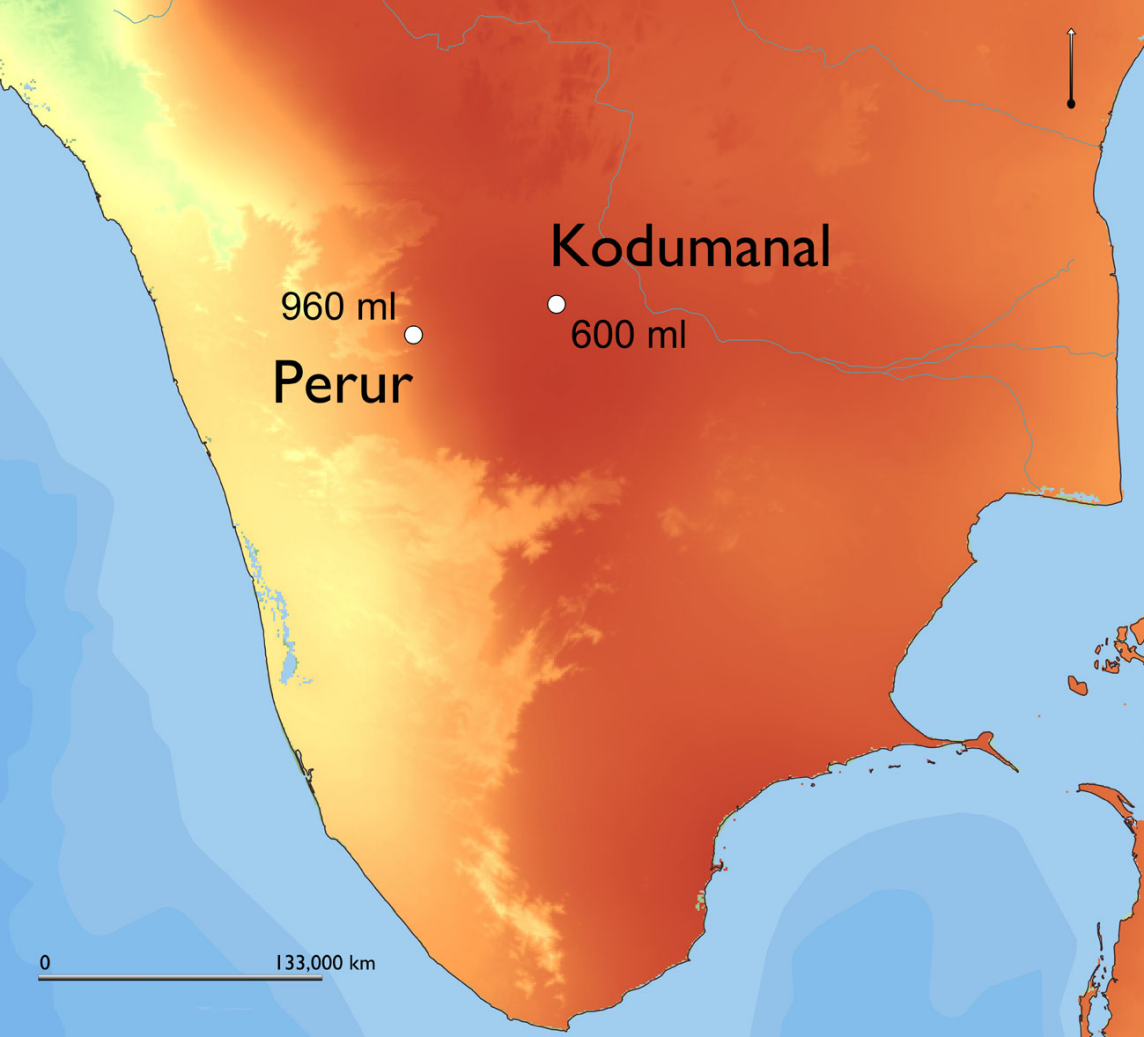
Map showing average annual rainfall levels at Perur and Kodumanal (based on the average monthly precipitation data in WorldClim v2 (Fick and Hijmans 2017))

As mentioned above, neither Neolithic-Chalcolithic Golbai Sasan or Tokwa had irrigation systems. The rice grown at both of these sites was therefore rainfed and so these samples represent flooded/decrue cultivation. This is consistent with the high quantity of dicotyledon phytoliths, representative of crop weeds, whose growth is suppressed in more intensively managed cultivation systems such as paddy fields by controlled and timed flooding (Caton et al. 2010; Weisskopf et al. 2013). Both sites are situated in low-lying areas next to rivers that see significant seasonal flooding with the monsoons, and this result is unsurprising. However, the samples from both Perur and Kodumanal are very dry in contrast. This data suggests that deep-water cultivation, and by extension, irrigation systems were not used for rice agriculture at Kodumanal or Perur c.500 BC–500 AD. Additionally, the samples from Kodumanal plot as slightly drier than those from Perur, reflecting rainwater availability (Fig. 5). This also supports the interpretation that rainfed cultivation was employed at these sites and suggests that the role of irrigation in the Iron Age-Early Historic South India should be re-examined.

### Reevaluating the evidence for irrigation in South India pre-500 AD

The modification of natural rock pools and reservoirs, in order to retain more water, has been identified at several Early Iron Age sites in Karnataka and (coupled to the recovery of rice, banana, wheat and barley) has been posited to represent the initial phase of the development of irrigation systems in South India, leading to tank, canal and dam construction in the Early Historic period (Bauer and Morrison 2008; Morrison, in press). However, it is important to question the likelihood that the altered natural ponds and reservoirs found across the central Southern Peninsula were primarily used for crop irrigation. They are often located on hill tops, without channels to feed water to agricultural fields, and, like Johansen and Bauer (2018), I would argue that a more likely agricultural use would be livestock watering. Pastoralism remains an important economy during the Iron Age. There is the suggestion that it saw a resurgence during the drier periods of the Early Iron Age based on the reoccupation of temporary hunting-pastoral sites such as Birappa post-1300 BC (Roberts et al. 2016; Shipton et al. 2012), and a decline in the number of sedentary settlement sites across the Southern Peninsula c.1200 BC (Roberts et al. 2016, p. 592). The value of having a protected and relatively secluded water source for watering herds of animals during a period of climatic fluctuations would have been high. This is especially true if there was risk of conflict with other groups (both mobile pastoralists and nearby settled pastoralists) during periods of water stress (see, for example Jia et al. 2017). If the hypothesis of protected hilltop settlement sites is expanded, associated megalithic features could potentially be seen as monuments delineating regional territories, constructed to be visible within the wider landscape. It is clear that water is symbolically important during the Iron Age, with the association of modified ponds with megalithic burial sites (Morrison 2015); however, the construction of monumental architecture is evidently incredibly nuanced (e.g. MacEachern and David 2013).

The argument that banana, wheat, barley and rice needed to be grown with supplemental watering provided by Iron Age reservoirs suggests that these crops were grown within an intensive, irrigated cultivation system (Morrison in press; Bauer and Morrison 2008). However, all of these crops could have been grown within other areas of seasonally waterlogged land, in a similar way to tank cultivation, without additional irrigation. In the absence of evidence for crop irrigation, and the work of Roberts et al. (2016) which shows a shift in settlement towards reliable rivers and alluvial plains, this low labour input scenario should be more readily considered. Prior to the development of agricultural irrigation in this region, larger and larger areas of seasonally flooded catchments could have been transformed into agricultural fields (extensification). It would be very interesting to see whether the larger Iron Age-Early Historic settlements tend to have been located next to larger areas of seasonally waterlogged land or not, and it is anticipated that the next decade of archaeological survey, settlement and landscape analysis will shed light on this.

The specific regional impacts of increased monsoon variability in the Late Holocene and more recent changes in regional climates also need to be considered in relation to settlement distribution and agricultural adaptation. Whilst it has been suggested that adopting or developing irrigation systems allowed for past societies to mitigate against fluctuating or decreasing rainfall levels in other parts of the world (e.g. Weber et al. 2010b; Castillo et al., 2019), it seems likely that in South India, mitigation strategies included maintaining crop diversity by cultivating a wide range of regionally adapted drought resilient millets, pulses and tubers.

### Rice as a symbolic crop in the Iron Age-Early Historic South India

**Fig. 6:**
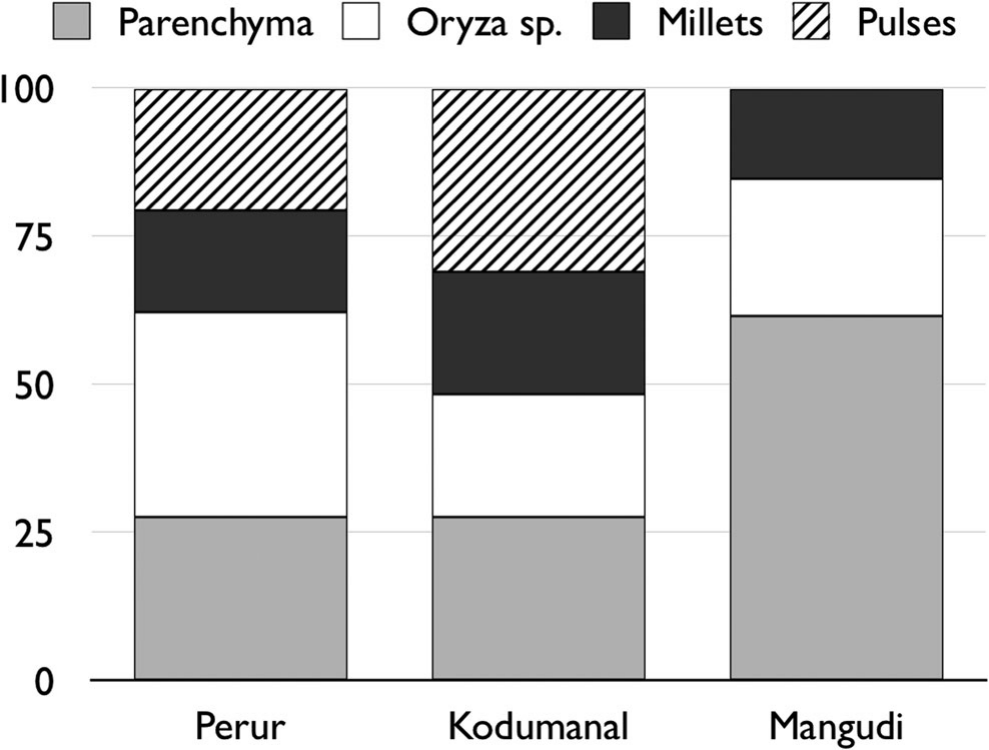
Relative abundance of main crop types identified by Cooke et al. (2005)

Without irrigation, yields of rice would have been lower than in other, wetter, areas of India, but it is unclear whether this holds significance for population growth and urban development. There is a limited amount of archaeobotanical data for the Iron Age-Early Historic South India (Table 1; in particular, note the large reduction in published data from 0 BC/AD), and so it is hard to assess the relative abundance of the different grain crops grown. The simple presence of rice does not mean that rice was the most frequently and abundantly consumed foodstuff. Where good archaeobotanical work has been done for this period, rice is shown to be a supplementary crop to tubers, millets and pulses (Cooke et al. 2005; Morrison et al. 2017) (Fig. 6). The data from the early urban sites of Mangudi and Kodumanal (Cooke et al. 2005) suggests that significant population growth was achieved and sustained via tuber, pulse and millet cultivation. In this case, South India can draw parallels with other regions of the world where millet and tubers were the primary crops, such as island Southeast Asia and West Africa (Fuller and Hildebrand 2013). Whether these tubers would have required irrigation is largely unknown. Taro (*Colocasia esculenta*) and a species of yam, *Dioscorea bulbifera*, are two tuber crops thought to be native to South Asia, and potentially domesticated here (Chaïr et al. 2016; Fuller 2006). Whilst they are largely grown under irrigation today, there are several records of drought tolerant cultivars, particularly cultivars grown in upland agricultural systems (Spencer 1988). Genetic diversity for taro is particularly high in Indian accessions (Chaïr et al. 2016; Ebert and Waqainabete 2018) supporting the possibility that a wider variety of drought tolerant tuber crops existed in the past (cf. Lebot 2009; Spencer 1988 pp. 111–5).

**Fig. 7:**
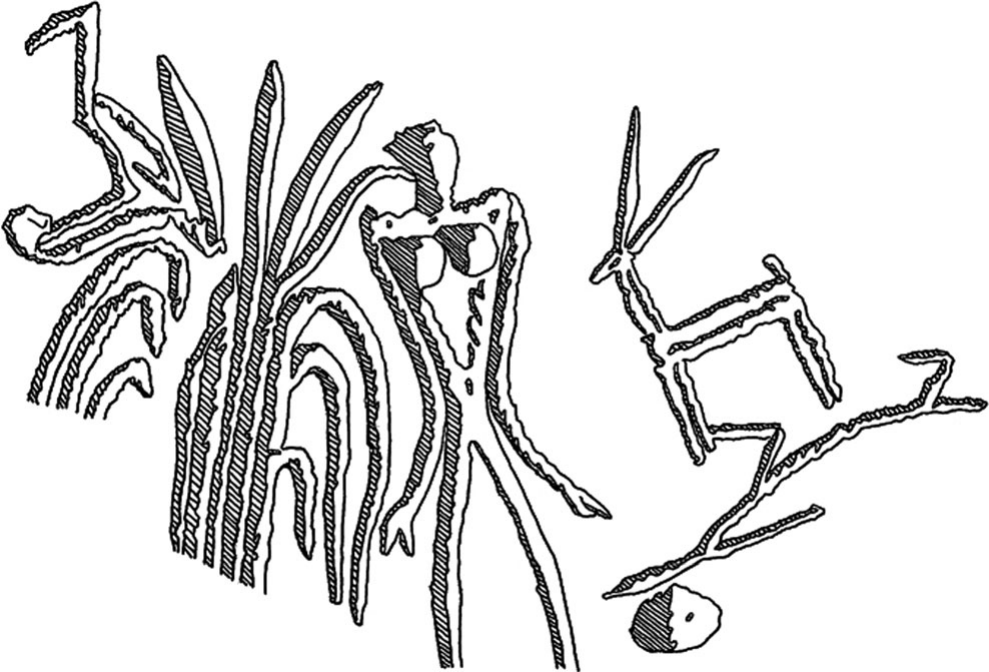
Motif from a pot fragment recovered from Adichanallur. (Drawing by D. Keshavarz, from an image reported by Subramanian (2005))

Large bulk finds of rice have been found dating to this period, however. Excavations at Porunthal cemetery uncovered several ceramic vessels filled with a large quantity of rice still in the husk, which has been radiocarbon dated to 490–450 BC (BETA 2018; Schug and Walimbe 2016 p. 331). These jars were recovered from burial contexts and have been interpreted as grave goods. No other crops were reported from the site. This discovery, as well as the similar discovery at Adichanallur, with urn burials dated to between 1500 and 400 BC (Sasisekaran et al. 2010), suggests that rice was a symbolic crop with a clear ceremonial use. Within this context, it is possible to think of rice as a special food, grown in small quantities for specific social occasions. The symbolic roles of rice across Asia are well documented (see for, e.g. Ammayao and Hamilton 2003), but the plant’s use as ‘social capital’ should also be considered in the context of South India (cf. Barton 2009; Madella 2014). It is possible that rice was grown initially in the Iron Age South India as a ‘boutique crop’ (as per Morrison in press, et al. 2017), alongside and perhaps equal to, sugarcane and banana, for specific ceremonial or social purposes (and with a high economic value), before becoming a more widely cultivated staple crop in the Historic period. The beautiful motif identified on pottery from Adichanallur appears to represent sugarcane (Fig. 7), hinting at the cultural significance of these boutique crops.

## Conclusion

Much more work needs to be done in order to accurately reconstruct the agricultural systems of ancient South Asia, but this study serves to demonstrate that a more nuanced and critical approach needs to be applied to discussions of agricultural systems in the Prehistoric and Early Historic South Asia. A ‘one size fits all’ theoretical model (in this case, ‘presence of rice = presence of irrigation’) can rarely be applied over such a large geographical and environmental gradient. The data presented in this study, although limited, casts some doubt over the notion that rice in South India was irrigated prior to 500 AD and has allowed for a discussion of alternative interpretations related to rice cultivation systems and the role of rice in this area. It is increasingly seems likely that there was a continuation in agricultural traditions in South India, from the Neolithic to well into the Early Historic period. This focused on the production of drought resilient crops (local small millets and pulses), with more water thirsty crops (including rice, cotton and sugar cane) the focus of more limited, but specialised, ‘boutique’ production.

As more archaeological survey and, crucially, more archaeobotanical work is conducted within South Asia, it is expected that increasing evidence will be found for regional variability within the Prehistoric and Early Historic South Asia, both within agricultural systems and related pathways to urbanisation. Within this context, and to allow our understanding to develop, more critical applications of the theories of intensification and the development of urban states must be incorporated into our analyses of South Asian archaeology.

## Electronic supplementary material

ESM 1 (XLSX 14 kb)

ESM 2 (XLSX 26 kb)

## References

[CR1] Abraham SA (2003). Chera, Chola, Pandya: using archaeological evidence to identify the Tamil kingdoms of Early Historic South India. Asian Perspect.

[CR2] Allchin FR (1995). The archaeology of early historic South Asia: the emergence of cities and states.

[CR3] Ames KM (1994). The northwest coast: complex hunter-gatherers, ecology, and social evolution. Annu Rev Anthropol.

[CR4] Ammayao A, Hamilton RW (2003). The art of rice: spirit and sustenance in Asia.

[CR5] Asouti E, Fuller DQ (2007). Trees and woodlands of South India: archaeological perspectives.

[CR6] Barton H (2009). The social landscape of rice within vegecultural systems in Borneo. Curr Anthropol.

[CR7] Bates J, Petrie CA, Singh RN (2017). Approaching rice domestication in South Asia: new evidence from Indus settlements in northern India. J Archaeol Sci.

[CR8] Bauer AM, Morrison KD, Selin H (2008). Water Management and Reservoirs in India and Sri Lanka. Encyclopaedia of the History of Science, Technology, and Medicine in Non-Western Cultures.

[CR9] BETA (Accessed 22 Jun 2018) How Porunthal’s Rice Grains Provided Insight to an Indian Writing System. http://www.radiocarbon.eu/carbon-dating-blog/767/porunthal-excavation/

[CR10] Bogaard A, Hodgson JG, Wilson PJ, Band SR (1998). An index of weed size for assessing the soil productivity of ancient crop fields. Veg Hist Archaeobotany.

[CR11] Bogaard A, Jones G, Charles M (2005). The impact of crop processing on the reconstruction of crop sowing time and cultivation intensity from archaeobotanical weed evidence. Veg Hist Archaeobotany.

[CR12] Boserup E (1965). The condition of agricultural growth. The economics of agrarian change under population pressure.

[CR13] Ter Braak C, Smilauer P (1998). CANOCO reference manual and User’s guide to Canoco for windows: software for canonical community ordination (version 4.5).

[CR14] Brookfield HC (1972). Intensification and disintensification in Pacific agriculture: a theoretical approach. Pacific Viewpoint.

[CR15] Castillo CC, Higham CFW, Miller K, Chang N, Douka K, Higham TFG, Fuller DQ (2019) Social responses to climate change in Iron Age Northeast Thailand: new archaeobotanical evidence. Antiquity

[CR16] Caton BP, Mortimer M, Hill JE, Johnson DE (2010). Weeds of rice in Asia.

[CR17] Chaïr H, Traore RE, Duval MF, Rivallan R, Mukherjee A, Aboagye LM, van Rensburg WJ, Andrianavalona V, Pinheiro de Carvalho MAA, Saborio F, Sri Prana M, Komolong B, Lawac F, Lebot V (2016). Genetic diversification and dispersal of taro (Colocasia esculenta (L.) Schott). PLoS One.

[CR18] Connah G (2001). African civilizations: an archaeological perspective.

[CR19] Cooke M, Fuller DQ (2015) Agricultural continuity and change during the Megalithic and Early Historic periods in South India. In: Basa KK, Mohanty RK, Ota SB (eds) Megalithic traditions in India. Archaeology and ethnography., 1st edn. Indira Gandhi Rashtriya Manav Sagrahalaya, Bhopal, pp 445–476

[CR20] Cooke M, Fuller DQ, Rajan K, Franke-Vogt U, Weisshaar H-J (2005). Early historic agriculture in southern Tamil Nadu: Archaeobotanical research at Mangudi, Kodumanal and Perur. South Asian archaeology 2003. Proceedings of the Seventeenth International Conference of the European Association of South Asian Archaeologists.

[CR21] Dictionary of Agriculture, Third edition (2011) A&C Black, London

[CR22] Ebert AW, Waqainabete LM (2018). Conserving and sharing taro genetic resources for the benefit of global taro cultivation: a core contribution of the centre for Pacific crops and trees. Biopreservation and Biobanking.

[CR23] Eichhorn B, Neumann K, Garnier A (2010). Seed phytoliths in West African Commelinaceae and their potential for palaeoecological studies. Palaeogeogr Palaeoclimatol Palaeoecol.

[CR24] Erickson CL, Marcus J, Stanish C (2006). Intensification, political economy, and the farming community; in defense of a bottom-up perspective of the past. Agricultural strategies.

[CR25] Fick SE, Hijmans RJ (2017). WorldClim 2: new 1-km spatial resolution climate surfaces for global land areas. Int J Climatol.

[CR26] Fitzhugh B (2003). The evolution of complex hunter-gatherers: archaeological evidence from the North Pacific.

[CR27] Fuller DQ (2001). Harappan seeds and agriculture: some considerations. Antiquity.

[CR28] Fuller DQ (2006). Agricultural origins and Frontiers in South Asia: a working synthesis. J World Prehist.

[CR29] Fuller DQ (2008) The spread of textile production and textile crops in India beyond the Harappan zone: an aspect of the emergence of craft specialization and systematic trade. In: Linguistics, archaeology and the human past. Occasoinal paper 3. Indus Project, Research Insitute for Humanity and Nature, pp 1–26

[CR30] Fuller Dorian, Hildebrand Elisabeth (2013). Domesticating Plants in Africa.

[CR31] Fuller DQ, Qin L (2009). Water management and labour in the origins and dispersal of Asian rice. World Archaeol.

[CR32] Fuller DQ, Sato Y-I, Castillo C, Qin L, Weisskopf AR, Kingwell-Banham EJ, Song J, Ahn S-M, van EJ (2010). Consilience of genetics and archaeobotany in the entangled history of rice. Archaeol Anthropol Sci.

[CR33] Fuller DQ, Weisskopf A, Castillo C (2016). Pathways of rice diversification across Asia. Archaeol Int.

[CR34] Fuller DQ, Castillo C, Kingwell-Banham E, Qin L, Weisskopf A, Fiorentino G, Zech-Matterne V (2017). Charred pummelo peel, historical linguistics and other tree crops: approaches to framing the historical context of early citrus cultivation in East, South and Southeast Asia. AGRUMED: Archaeology and history of citrus fruit in the Mediterranean : Acclimatization, diversifications, uses.

[CR35] Gilliland K, Simpson IA, Adderley WP, Burbidge CI, Cresswell AJ, Sanderson DCW, Coningham RAE, Manuel M, Strickland K, Gunawardhana P, Adikari G (2013). The dry tank: development and disuse of water management infrastructure in the Anuradhapura hinterland, Sri Lanka. J Archaeol Sci.

[CR36] Guha R (1999). Elementary aspects of peasant insurgency in colonial India.

[CR37] Hall KR, Hall KR (2001). Merchants, rulers, and priests in an early south Indian sacred center: Cidabaram in the age of the colas. Structure and society in Early South India: essays in honor of Noboru Karashima.

[CR38] Harvey EL (2006) Early agricultural communities in northern and eastern India: an archaeobotanical investigation. Doctoral thesis, University of London

[CR39] Huan X, Lu H, Zhang J, Wang C (2018). Phytolith assemblage analysis for the identification of rice paddy. Sci Rep.

[CR40] IRRI (2000). Rainfed Rice: a Sourcebook of Best Practices and Strategies in Eastern India.

[CR41] Jenkins E, Jamjoum K, Nuimat S, Mithen S, Black E (2011). Irrigation and phytolith formation: an experimental study. Water, life and civilisation: climate, Environment and Society in the Jordan Valley.

[CR42] Jenkins E, Jamjoum K, Nuimat S, Stafford R, Nortcliff S, Mithen S (2016). Identifying ancient water availability through phytolith analysis: an experimental approach. J Archaeol Sci.

[CR43] Jia PW, Betts A, Doumani Dupuy PN, Cong D, Jia X (2017). Bronze Age Hill Forts: new evidence for defensive sites in the western Tian Shan, China. Archaeological Research in Asia.

[CR44] Johansen PG (2010). Site maintenance practices and settlement social organization in Iron Age Karnataka, India: inferring settlement places and landscape from surface distributions of ceramic assemblage attributes. J Anthropol Archaeol.

[CR45] Johansen PG, Bauer AM (2018). On the matter of resources and techno-politics: the case of water and iron in the South Indian Iron Age. Am Anthropol.

[CR46] Jones M (2009). Dormancy and the plough: weed seed biology as an indicator of agrarian change in the first millennium AD. From foragers to farmers: papers in honour of Gordon C. Hillman.

[CR47] Jones G, Bogaard A, Charles M, Hodgson JG (2000). Distinguishing the effects of agricultural practices relating to fertility and disturbance: a functional ecological approach in archaeobotany. J Archaeol Sci.

[CR48] Kingwell-Banham EJ (2015) Early rice agriculture in South Asia. Identifying cultivation systems using archaeobotany. Doctoral thesis, UCL (University College London)

[CR49] Kingwell-Banham E, Fuller DQ (2012). Shifting cultivators in South Asia: expansion, marginalisation and specialisation over the long term. Quat Int.

[CR50] Kingwell-Banham E, Petrie CA, Fuller DQ, Barker G, Goucher C (2015). Early agriculture in South Asia. The Cambridge world history.

[CR51] Kingwell-Banham E, Karoune E, Mohanty RK, Fuller DQ (2018). Golbai Sasan and Gopalpur. Neolithic-Chalcolithic settlements of Odisha and their archaeobotanical remains. Ancient Asia.

[CR52] Kirch PV (1995). The wet and the dry: irrigation and agricultural intensification in Polynesia.

[CR53] Köppen WP (1923). Die Klimate der Erde: Grundriss der Klimakunde.

[CR54] Lebot V (2009). Tropical root and tuber crops : cassava, sweet potato, yams and aroids.

[CR55] Liu L (2005). The Chinese Neolithic: trajectories to early states.

[CR56] Lu H, Zhang J, Wu N, Liu K, Xu D, Li Q (2009). Phytoliths analysis for the discrimination of foxtail millet (Setaria italica) and common millet (Panicum miliaceum). PLoS One.

[CR57] MacEachern S, David N (2013). Monumental architecture in mountain landscapes: the diy-geδ-bay sites of northern Cameroon. Azania: Archaeol Res Africa.

[CR58] Madella M (2014) Of crops and food:: a social perspective on rice in the Indus Civilization. In: Madella M, Lancelotti C, Savard M (eds) Ancient plants and people. University of Arizona Press, pp 218–236

[CR59] Madella M, Jones MK, Echlin P, Powers-Jones A, Moore M (2009). Plant water availability and analytical microscopy of phytoliths: implications for ancient irrigation in arid zones. Quat Int.

[CR60] Marcus J, Stanish C (2006). Agricultural strategies.

[CR61] Marquet PA, Santoro CM, Latorre C, Standen VG, Abades SR, Rivadeneira MM, Arriaza B, Hochberg ME (2012). Emergence of social complexity among coastal hunter-gatherers in the Atacama Desert of northern Chile. PNAS.

[CR62] Marston JM (2011). Archaeological markers of agricultural risk management. J Anthropol Archaeol.

[CR63] McClatchie M, Smith C (2014). Archaeobotany of agricultural intensification. Encyclopedia of global archaeology.

[CR64] Miller HML, Marcus J, Stanish C (2006). Water supply, labor requirements, and land ownership in Indus floodplain agricultural systems. Agricultural Strategies.

[CR65] Misra VD, Pal JN, Gupta MC (2000). Excavation at Tokwa: a Neolithic-Chalcolithic settlement. Pragdhara.

[CR66] Moorti US (1994). *Megalithic Culture of South India: Socio-economic Perspectives*.

[CR67] Morrison KD (1994). The intensification of production: archaeological approaches. J Archaeol Method Theory.

[CR68] Morrison KD, Petraglia MD, Allchin B (2007). Foragers and forager-traders in South Asian worlds: some thoughts from the last 10,000 years. The evolution and history of human populations in South Asia.

[CR69] Morrison KD (2015). Archaeologies of flow: water and the landscapes of southern India past, present, and future. J Field Archaeol.

[CR70] Morrison KD (2019) *Water in South India and Sri Lanka: Agriculture, Irrigation, Politics, and Purity*. In: Yasuda Y, Scarborough V (eds) History of water and civilization, volume VII, water and humanity: an historical overview. UNESCO publishing

[CR71] Morrison KD, Junker LL (2002). Forager-traders in south and Southeast Asia long-term histories.

[CR72] Morrison KD, Reddy SN, Kashyap A (2017) *Agrarian Transitions in Iron Age South India: Social and Environmental Implications.* In: South Asian archaeology and art 2012: man and environment in prehistoric and protohistoric South Asia: new perspectives (Indicopleustoi, Achaeologies of the Indian Ocean). Brepols Pub, pp 185–196

[CR73] Mosse D (1999). Colonial and contemporary ideologies of ‘community management’: the case of tank irrigation development in South India. Mod Asian Stud.

[CR74] Murphy CA, Fuller DQ, Schug GR, Walimbe SR (2016). The transition to agricultural production in India: south Asian entanglements of domestication. A Companion to South Asia in the past.

[CR75] Patnaik R, Gupta AK, Naidu PD, Yadav RR, Bhattacharyya A, Kumar M (2012). Indian monsoon variability at different time scales: marine and terrestrial proxy records. Proc Indian Natl Sci Acad.

[CR76] Penny D, Kealhofer L (2005). Microfossil evidence of land-use intensification in North Thailand. J Archaeol Sci.

[CR77] Petrie CA, Bates J (2017). ‘Multi-cropping’, intercropping and adaptation to variable environments in Indus South Asia. J World Prehist.

[CR78] Piperno DR (2006). Phytoliths: a comprehensive guide for archaeologists and paleoecologists.

[CR79] Pokharia AK (2008). Palaeoethnobotanical record of cultivated crops and associated weeds and wild taxa from Neolithic site, Tokwa, Uttar Pradesh, India. Curr Sci.

[CR80] Ponton C, Giosan L, Eglinton TI, Fuller DQ, Johnson JE, Kumar P, Collett TS (2012). Holocene aridification of India. Geophys Res Lett.

[CR81] Porter BW, Routledge BE, Simmons EM, Lev-Tov JSE (2014). Extensification in a Mediterranean semi-arid marginal zone: an archaeological case study from Early Iron Age Jordan’s Eastern Karak Plateau. J Arid Environ.

[CR82] Prasad S, Anoop A, Riedel N, Sarkar S, Menzel P, Basavaiah N, Krishnan R, Fuller D, Plessen B, Gaye B, Röhl U, Wilkes H, Sachse D, Sawant R, Wiesner MG, Stebich M (2014). Prolonged monsoon droughts and links to Indo-Pacific warm pool: a Holocene record from Lonar Lake, Central India. Earth Planet Sci Lett.

[CR83] Raman KV (2008) Irrigation in South India (up to 1300 AD): techniques and management. In Gopal L and Srivastava VC (eds.) History of science, philosophy and culture in Indian civilization. Volume 5, part 1. History of Agriculture in India (up to c.1200 AD). PHISPC Centre for studies in civilizations, India, pp 496–505

[CR84] Ramaswamy V (2008) The history of agriculture in South India. In Gopal L and Srivastava VC (eds.) History of science, philosophy and culture in Indian civilization. Volume 5, part 1. History of Agriculture in India (up to c.1200 AD). PHISPC Centre for studies in civilizations, India, pp 617–639

[CR85] Roberts P, Boivin N, Petraglia M, Masser P, Meece S, Weisskopf A, Silva F, Korisettar R, Fuller DQ (2016). Local diversity in settlement, demography and subsistence across the southern Indian Neolithic-Iron Age transition: site growth and abandonment at Sanganakallu-Kupgal. Archaeol Anthropol Sci.

[CR86] Rosen AM, Weiner S (1994). Identifying ancient irrigation: a new method using opaline phytoliths from emmer wheat. J Archaeol Sci.

[CR87] Sasisekaran B, Sundararajan S, Venhata Rao D, Raghunatha Roa B, Badrinarayanan S, Rajavel S (2010). Adichanallur: a prehistoric mining site. Indian J Hist Sci.

[CR88] Schug GR, Walimbe SR (2016) A companion to South Asia in the past. John Wiley & Sons

[CR89] Scott JC (2009). The art of not being governed: an anarchist history of upland Southeast Asia.

[CR90] Scott JC (2017). Against the grain. A deep history of the earliest states.

[CR91] Sengupta S (2000). Political economy of irrigation: tanks in Orissa, 1850-1996. Econ Polit Wkly.

[CR92] Shaw J (2005) Landscape, water and religion in ancient India. Archaeol Int 9. 10.5334/ai.0912

[CR93] Shaw J, Sutcliffe J (2003). Ancient dams, settlement archaeology and Buddhist propagation in Central India: the hydrological background. Hydrol Sci J.

[CR94] Shaw J, Sutcliffe J, Lloyd-Smith L, Schwenninger J-L, Chauhan MS (2007). Ancient irrigation and Buddhist history in Central India: optically stimulated luminescence dates and pollen sequences from the Sanchi dams. Asian Perspect.

[CR95] Shipton C, Petraglia M, Koshy J, Bora J, Brumm A, Boivin N, Korisettar R, Risch R, Fuller DQ (2012). Lithic technology and social transformations in the South Indian Neolithic: the evidence from Sanganakallu–Kupgal. J Anthropol Archaeol.

[CR96] Silva Fabio, Weisskopf Alison, Castillo Cristina, Murphy Charlene, Kingwell-Banham Eleanor, Qin Ling, Fuller Dorian Q (2018). A tale of two rice varieties: Modelling the prehistoric dispersals of japonica and proto-indica rices. The Holocene.

[CR97] Smith ML (2006). The archaeology of South Asian cities. J Archaeol Res.

[CR98] Spencer JE (1988). Shifting cultivation in southeastern Asia.

[CR99] Stargardt J, Zhuang Y, Altaweel M (2019). Water for the state or water for the people? Wittfogel in South and South East Asia in the first millennium. Water Societies and Technologies from the Past and Present.

[CR100] Stevens C, Murphy C, Roberts R, Lucas L, Silva F, Fuller D (2016). Between China and South Asia: a middle Asian corridor of crop dispersal and agricultural innovation in the Bronze Age. The Holocene.

[CR101] Stone GD (1996). Settlement ecology: the social and spatial organization of Kofyar Agriculture.

[CR102] Styring AK, Charles M, Fantone F, Hald MM, McMahon A, Meadow RH, Nicholls GK, Patel AK, Pitre MC, Smith A, Sołtysiak A, Stein G, Weber JA, Weiss H, Bogaard A (2017). Isotope evidence for agricultural extensification reveals how the world’s first cities were fed. Nature Plants.

[CR103] Subramanian TS (2005) Unearthing a great past. Frontline 22(13)Jun18-Jul01. https://www.frontline.in/static/html/fl2213/stories/20050701000106500.htm

[CR104] Tewari R, Srivastava RK, Saraswat KS, Singh IB, Singh KK (2008). Early farming at Lahuradewa. Pragdhara.

[CR105] Thakur R (2002). Mechanisms of urban growth in India: AD 600–1200. Urban History.

[CR106] Thapar R (2004). Early India: from the origins to A.D. 1300.

[CR107] Tripathi S, Basumatary SK, Singh VK, Bera SK, Nautiyal CM, Thakur B (2014). Palaeovegetation and climate oscillation of western Odisha, India: a pollen data-based synthesis for the Mid-Late Holocene. Quat Int.

[CR108] Weber SA (1998). Out of Africa: the initial impact of millets in South Asia. Curr Anthropol.

[CR109] Weber S (1999). Seeds of urbanism: palaeoethnobotany and the Indus Civilization. Antiquity.

[CR110] Weber S, Kashyap A, Harriman D (2010). Does size matter: the role and significance of cereal grains in the Indus civilization. Archaeol Anthropol Sci.

[CR111] Weber S, Lehman H, Barela T, Hawks S, Harriman D (2010). Rice or millets: early farming strategies in prehistoric Central Thailand. Archaeol Anthropol Sci.

[CR112] Weiss H, Weiss H (1986). The origins of Tell Leilan and the conquest of space in third millennium Mesopotamia. The Origins of cities in dry-farming Syria and Mesopotamia in the third millennium B.C..

[CR113] Weisskopf A (2017). A wet and dry story: distinguishing rice and millet arable systems using phytoliths. Veg Hist Archaeobotany.

[CR114] Weisskopf A, Harvey E, Kingwell-Banham E, Kajale M, Mohanty R, Fuller DQ (2013). Archaeobotanical implications of phytolith assemblages from cultivated rice systems, wild rice stands and macro-regional patterns. J Archaeol Sci.

[CR115] Weisskopf A, Qin L, Ding J, Ding P, Sun G, Fuller DQ (2015). Phytoliths and rice: from wet to dry and back again in the Neolithic lower Yangtze. Antiquity.

[CR116] Wittfogel K (1957). Oriental despotism: a comparative study of total power.

[CR117] Zhuang Y, Ding P, French C (2014). Water management and agricultural intensification of rice farming at the late-Neolithic site of Maoshan, Lower Yangtze River, China. The Holocene.

